# The effect of fuel on the physiochemical properties of ZnFe_2_O_4_ synthesized by solution combustion method

**DOI:** 10.55730/1300-0527.3487

**Published:** 2022-07-21

**Authors:** Fahma RIYANTI, Widia PURWANINGRUM, Nova YULIASARI, Sasmita PUTRI, Nabila APRIANTI, Poedji Loekitowati HARIANI

**Affiliations:** 1 Department of Chemistry, Faculty of Mathematics and Natural Sciences, Sriwijaya University, Indralaya, Indonesia; 2 Doctoral Program of Environmental Science, Graduate School, Sriwijaya University, South Sumatra, Indonesia

**Keywords:** Solution combustion, ZnFe_2_O_4_, urea, glycine, EDTA, physicochemical

## Abstract

The synthesis of ZnFe_2_O_4_ nanoparticles was performed using the solution combustion method with three types of fuel, namely urea, glycine, and ethylenediamine tetra-acetic acid (EDTA) with precursors (Zn(NO_3_)_2_.6H_2_O and Fe(NO_3_)_3_.9H_2_O. The combustion process was conducted in an open space at 300 °C for ± 1 h, resulting in a brownish-black ZnFe_2_O_4_. Meanwhile, the fuel type used in the process affects the physicochemical properties of ZnFe_2_O_4_. XRD analysis showed that ZnFe_2_O_4_ synthesized using urea, glycine, and EDTA had spinel structures with crystal sizes of 10.19, 20.34, and 27.21 nm, respectively. The FTIR spectra of ZnFe_2_O_4_ synthesized using the three fuel types had Zn-O and Fe-O stretching vibrations. Furthermore, the morphology of ZnFe_2_O_4_ synthesized using urea was more homogeneous than glycine and EDTA. The saturation magnetization of ZnFe_2_O_4_ synthesized using EDTA was 54.63 emu/g compared to glycine and urea, 50.93 and 44.73 emu/g, respectively. Finally, the surface area of synthesized ZnFe_2_O_4_ using urea, glycine, and EDTA were 116.4, 100.6, and 94.2 m^2^/g, respectively.

## 1. Introduction

In recent years, nanosized materials have been examined intensively. Furthermore, nanotechnology is the science of technology, referring to the ability to engineer and utilize materials as well as devices with dimensions between 1 and 100 nm [1]. Nanosized materials have unique chemical and physical properties compared to the bulk form [2]. Meanwhile, nanotechnology is becoming increasingly influential in various fields of application, ranging from the environment, to the food industry, to development even in the biomedical field, showing great potential for future clinics [3]. For example, ferrite is a magnetic nanoparticle characterized by a spinel structure with the general formula of MFe_2_O_4_, where M and Fe are metal cations located at the tetrahedral and octahedral sites [4]. Zinc ferrite (ZnFe_2_O_4_) is an important compound widely used in various industrial applications, such as gas sensors [5], batteries [6], catalysts [7,8], and adsorbents [9,10].

The synthesis method used influences the properties of ferrite compounds, including the size, shape, morphology, surface area, and magnetic properties [11]. Several methods of synthesizing ZnFe_2_O_4_ have been reported, including ball milling [12], coprecipitation [13,14], sol-gel [15], hydrothermal [16], and solution combustion [17]. Furthermore, this method has disadvantages, such as the formation of unwanted phases, complexity, and high cost. Therefore, a simple, easy, and low-cost technique is needed.

Solution combustion is a high-temperature synthesis that is effective and inexpensive for preparing nanomaterials such as ferrite, perovskite, and zirconia [11]. In addition, the reaction requires fast time (a few minutes) and simple equipment [18,19]. This method involves an independent reaction between an oxidizing agent (e.g., metal nitrate) as a precursor salt and a fuel (e.g., EDTA, glycine, hydrazine, urea, citric acid) [11,20]. The reactants are dissolved in water until it becomes homogeneous. Furthermore, it is heated to the boiling point of the medium, and evaporation occurs. The solution is ignited or self-ignites as the temperature rises rapidly. Simultaneously, the mixed solution changes into a fine crystalline powder of the desired composition [11]. In this process, a redox reaction or electron transfer occurs, oxidizing the fuel, and the oxidizing agent is reduced, leading to an exothermic reaction [21].

The type of fuel used in the solution combustion affects the phase formation and morphology of the resulting nanomaterial [22]. Several fuels being used include urea, glycine, oxalyldihydrazine, carbohydrazide, EDTA, citric acid, and sucrose [23,24]. The synthesis of metal oxides using the method with several types of fuel has been examined, such as Fe_3_O_4_ using glycine [25], Bi_2_O_3_ using urea, glycine, and citric acid [26], NiO using urea and glycine [22], and NiFe_2_O_4_ using urea. [27]. Meanwhile, there is no detailed information on the suitable fuel type to synthesize specific nanomaterials. For example, the synthesis of nanomagnetic NiFe_2_O_4_ using fuel containing nitrogen (urea) produces a larger particle size than those from the hydrocarbon group [23]. This fuel type produces a variety of combustion, ranging from mild reactions that only produce mass to intense combustion reactions, which result in intense flames and explosions [28,29]. Therefore, this research aimed to explore the synthesis of ZnFe_2_O_4_ using fuel types, namely urea, glycine, and EDTA, and its effect on crystal size, magnetic properties, and surface area. The characteristics were analyzed using XRD, FTIR, SEM-EDS, and specific surface area with BET.

## 2. Materials and methods

### 2.1. Materials

The chemicals used include Zn(NO_3_)_2_.6H_2_O, Fe(NO_3_)_3_.9H_2_O, KCl, CH_4_N_2_O (urea), NH_2_CH_2_COOH (glycine), and C_10_H_16_N_2_O_8_ (EDTA), and were purchased from Merck Company. Also, distilled water was used for the experiment.

### 2.2. Synthesis of ZnFe_2_O_4_ Using the Solution Combustion Method

The synthesis procedure of ZnFe_2_O_4_ was as follows: 60 mL of distilled water was added to 3 beakers of 250 mL. Then, 0.5 M HNO_3_ was slowly added until it reached pH 4. A total of 3.336 g Glycine, 4.0 g Urea, and 5.844 g EDTA were added to each beaker, 2.975 g of Zn(NO_3_)_2_.6H_2_O was added and stirred slowly for 10 min. Furthermore, 8.080 g of Fe(NO_3_)_3_.9H_2_O and 1.4919 g KCl were added in quantity. The mixture was homogenized using a stirrer for 15 min at room temperature. Continuously, it was stirred with a magnetic stirrer at 300 °C. After the solution changed color and the combustion process occurred, the stirring was stopped. It was further heated at 300 °C until a complete combustion reaction (±1 h). Finally, the resulting product was powder, washed with 200 mL of boiling distilled water, and dried in an oven at 80 °C for 1 h.

### 2.3. ZnFe_2_O_4_ characterization

The crystal structure and phase were analyzed using an X-ray diffractometer (XRD Shimadzu 7000 diffractometer) at Cu-Kα radiation = 1548 Å and range 2θ = 10–80º. The following Debye Scherrer equation (Eq. 1) was used to determine crystal size [30]:


(1) $$D=\frac{k\lambda }{\beta \;\text{cos }\theta },$$

where D is the average crystal size of ZnFe_2_O_4_, λ is the X-ray wavelength (0.15418 nm), k is the Scherrer constant (0.9), β is full width at half maximum (FWHM), and θ is the Bragg diffraction angle.

The functional groups were analyzed using Fourier transform infrared (FT-IR Prestige 21 Shimadzu) at a wavenumber of 500–4000 cm^−1^. Meanwhile, magnetic properties were analyzed using a vibrating sample magnetometer (VSM Lakeshore 74004) at room temperature, and the surface area was analyzed using the ASAP 2020.

## 3. Results and discussion

**Figure 1:**
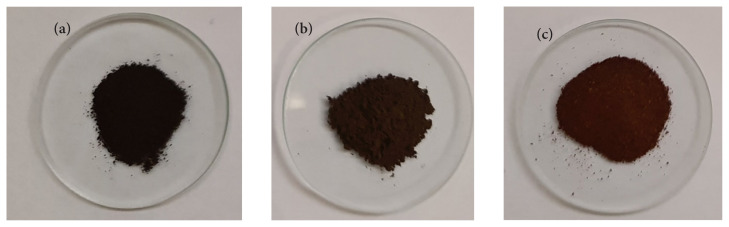
ZnFe_2_O_4_ synthesized using (a) urea (b) glycine and (c) EDTA.

Figure 1 shows the synthesized ZnFe_2_O_4_ using the solution combustion method with various fuel types, namely urea, glycine and EDTA. The reaction product is a brownish-black ZnFe_2_O_4_ powder as well as H_2_O, CO_2_, and N_2_ gases. The following shows the reaction of ZnFe_2_O_4_ synthesis using urea, glycine, and EDTA as fuel:


$$\begin{array}{l} 3\text{ZN}{\left({\text{NO}}_{3}\right)}_{2}.6{\text{H}}_{2}\text{O}+6\text{Fe}{\left({\text{NO}}_{3}\right)}_{3}.9{\text{H}}_{2}\text{O}+20{\left({\text{NH}}_{2}\right)}_{2}\text{CO}\to 3{\text{ZnFe}}_{2}{\text{O}}_{4}+112{\text{H}}_{2}\text{O}+20{\text{CO}}_{2}+32{\text{N}}_{2}\\ 9\text{ZN}{\left({\text{NO}}_{3}\right)}_{2}.6{\text{H}}_{2}\text{O}+18\text{Fe}{\left({\text{NO}}_{3}\right)}_{3}.9{\text{H}}_{2}\text{O}+40{\text{C}}_{2}{\text{H}}_{5}{\text{NO}}_{2}\to 9{\text{ZnFe}}_{2}{\text{O}}_{4}+316{\text{H}}_{2}0+80{\text{CO}}_{2}+56{\text{N}}_{2}\\ \text{ZN}{\left({\text{NO}}_{3}\right)}_{2}.6{\text{H}}_{2}\text{O}+2\text{Fe}{\left({\text{NO}}_{3}\right)}_{3}.9{\text{H}}_{2}\text{O}+{\text{C}}_{10}{\text{H}}_{16}{\text{N}}_{2}{\text{O}}_{8}\to {\text{ZnFe}}_{2}{\text{O}}_{4}+32{\text{H}}_{2}0+10{\text{CO}}_{2}+5{\text{N}}_{2}. \end{array}$$

Metal nitrate is often used as an oxidizing agent because it has a higher solubility (approximately 64%) than sulfate (approximately 27%) [31]. The ideal fuel needs to have a high solubility in solvents, such as water, a low decomposition temperature (below 400 °C), produce no other residual mass, and be compatible with metal nitrates. However, other solvents such as alcohol and kerosene are used [32,33]. Maximum energy is released when the reaction is in a stoichiometric state. An oxygen supply is needed to achieve complete combustion [18]. In this research, the combustion reaction was performed in an open space at 200–300 ºC, with the contribution of oxygen in the atmosphere [26]. The addition of KCl reduces the crystal size and increases the surface area. The higher addition of KCl and NaCl in the synthesis of ZnFe_2_O_4_ using the solution combustion method with L-α Alanine as fuel decreases the crystal size and increases the surface area [34].

**Figure 2:**
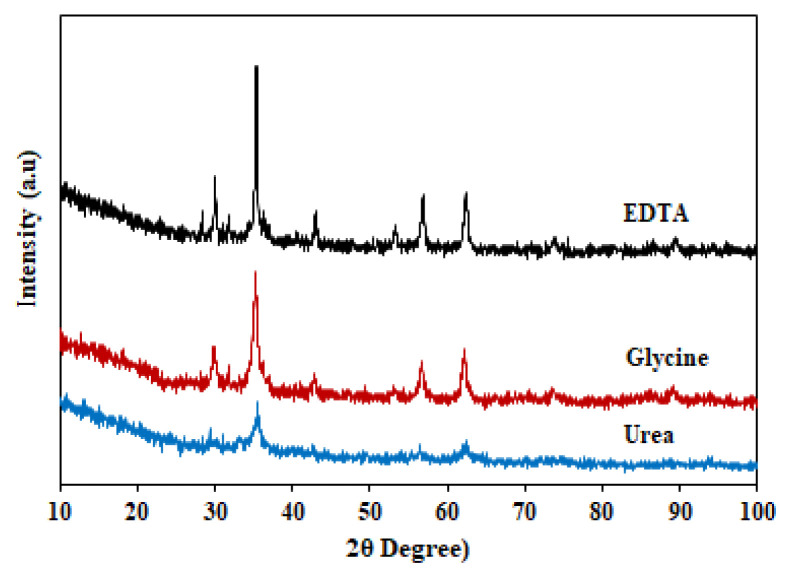
XRD spectra of ZnFe_2_O_4_ with fuel (a) urea (b) glycine (c) EDTA.

According to JCPDS No. 22-1012, ZnFe_2_O_4_ has a spinel structure, which is at 2θ = 29.97°, 35.29°, 42.91°, 56.75°, and 62.32°, where the plane index (220), (311), (400), (511), and (440) is a plane cubic (Figure 2). Therefore, the type of fuel used in the synthesis of ZnFe_2_O_4_ affects the peak intensity of the XRD spectra. Furthermore, the highest peak intensity indicating greater crystallinity was observed in ZnFe_2_O_4_ synthesized using EDTA. The crystal size of ZnFe_2_O_4_ synthesized using urea, glycine, and EDTA was 10.19, 20.34, and 27.21 nm, respectively (Table 1).

The fuel’s chain length (molecular weight) affects the crystallinity, crystal size, and particle size. It is also related to the solubility and complexation of fuel. Fuels with longer molecular chains produce a large amount of gas released during the process. In addition, fuels with a larger molecular mass have more sites for metal cations’ complex formation and solubility [35]. EDTA has a molecular mass (Mw = 336.21 g/mol), greater than urea (Mw = 60.05 g/mol) and glycine (Mw = 75.07 g/mol). Another factor is the bonding heat of the reaction, depending on the number of single and double bonds in the fuel. The double bond fuel, such as urea (triple), are called unsaturated bonds and are generally more reactive. Therefore, the crystal formation process occurs quicker [26,34].

**Figure 3:**
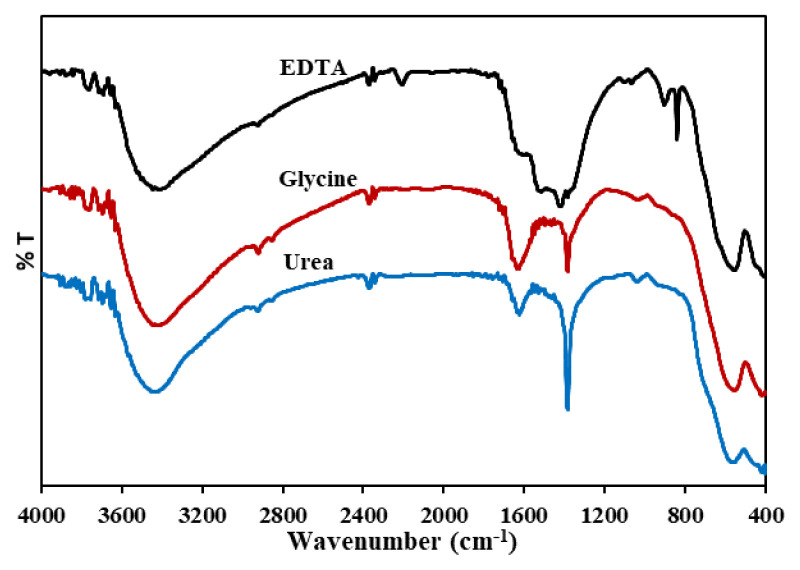
Spectra FTIR of ZnFe_2_O_4_ synthesized using (a) urea, (b) glycine, (c) EDTA.

Figure 3 shows the FTIR spectra of ZnFe_2_O_4_ synthesized using urea, glycine, and EDTA. The wavenumber at 3200–3600 cm^−1^ is the stretching vibration of the O-H functional group. Furthermore, the presence of this functional group is enhanced by absorption at a wavenumber of approximately 1650 cm^−1,^ which is a stretch bending of O-H [36,37]. This absorption was observed in ZnFe_2_O_4_ synthesized using glycine and EDTA. Two absorption bands at wave numbers approximately 550 cm^−1^ and 430 cm^−1^ are stretching vibrations of Zn-O and Fe-O bonds, namely the tetrahedral and the octahedral sites [38]. The wavenumbers appear at 557.43 and 416.62 cm^−1^ (fuel: urea), 553.57 and 408.9 (fuel: glycine), as well as 553.57 and 410.83 cm^−1^ (fuel: EDTA). The presence of wavenumber at 1300 cm^−1^ indicates a C=O group of the remaining fuel.

**Figure 4:**
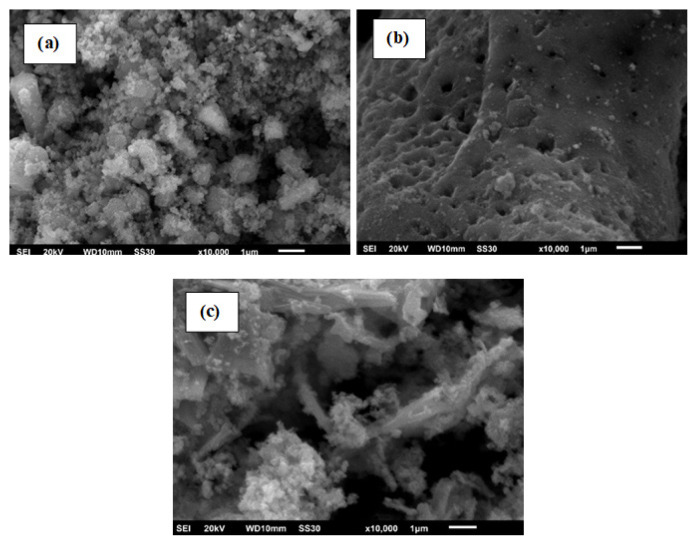
The morphology of ZnFe_2_O_4_ synthesized using (a) urea, (b) glycine, and (c) EDTA.

Figure 4 shows the morphology of ZnFe_2_O_4_ synthesized using urea, glycine, and EDTA The morphology of ZnFe_2_O_4_ synthesized with urea fuel appears more homogeneous and has a smaller particle size than with glycine and EDTA. On the other hand, ZnFe_2_O_4_ synthesized using glycine fuel appears as large and porous crystals. The results are similar to the synthesis of Bi_2_O_3_ using glycine, which has an elliptical and porous structure [26,39].

Table 2 shows the percentage of elements in ZnFe_2_O_4_ due to the analysis using EDS. ZnFe_2_O_4_ synthesized using different fuel types contains the same elements, namely Zn, O, and Fe, with different percentages. Furthermore, the stoichiometric content of these elements is 27.13%, 46.33%, and 26.54%. A similar composition was observed in ZnFe_2_O_4_ synthesized using urea.

The surface area affects the increase and decrease in the magnetic properties of nanoparticles. For example, it was reported that the magnetization of the oxide nanoparticles decreases in direct proportion to the particle size [40]. In contrast, the magnetization of some metal (cobalt) nanoparticles were reported to increase directly to particle size [41]. The decrease in magnetization of oxide nanoparticles is caused by the presence of a magnetic dead layer on the particles’ surface due to the spin-glass-like behavior [40].

**Figure 5:**
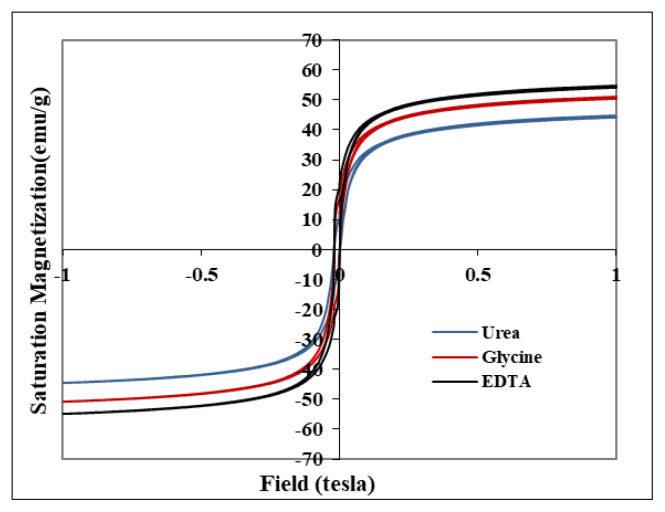
Magnetization curve of ZnFe_2_O_4_ synthesized using (a) urea, (b) glycine, and (c) EDTA.

The nanoparticle synthesis method is essential in determining the shape, particle size, size distribution, and surface chemistry of the particles, thereby determining their magnetic properties [42,43]. In this research, ZnFe_2_O_4_ synthesized using urea, glycine, and EDTA had saturation magnetization of 44.72, 50.93, and 54.63 emu/g, respectively, proportional to the particle size (Figure 5). According to Li et al.’s [18] study on the synthesis of Fe_3_O_4_, the values of coercivity (Hc), remanent magnetization (Mr), and saturation magnetization (Ms) increased with increasing particle size to a maximum value which later becomes constant or decreased. Therefore, there should be a good balance between effective surface area and satisfactory magnetic performance [18,44]. When the nanoparticle size is small enough, it has superparamagnetic properties and responds mainly to the applied magnetic field [45].

Another research showed that ZnFe_2_O_4_ synthesized using the solvothermal method at various times resulted in increased crystal size and increased magnetic properties [46]. Table 3 shows the results of surface area measurements of ZnFe_2_O_4_ synthesized using urea, glycine, and EDTA of 116.4, 100.6, and 94.2 m^2^/g, respectively. ZnFe_2_O_4_ synthesized using urea has the largest surface area of glycine and EDTA fuels.

**Figure 6:**
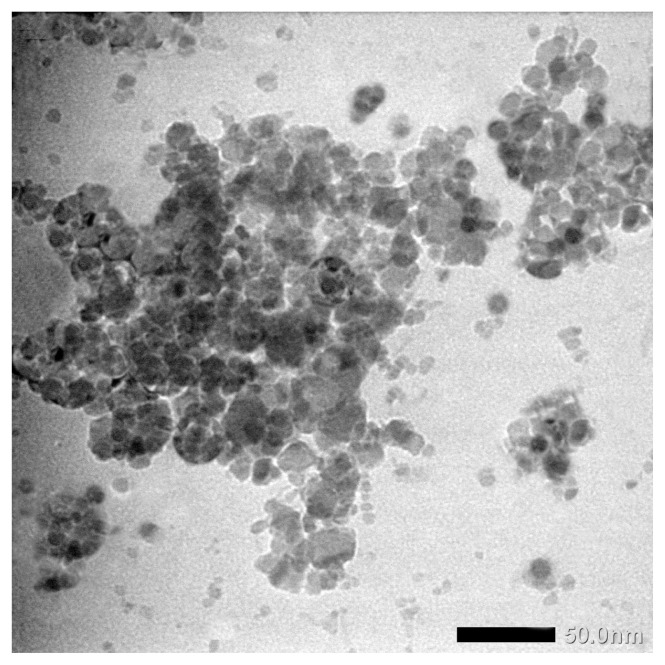
TEM image of ZnFe_2_O_4_ synthesized using urea.

Figure 6 shows a TEM image of ZnFe_2_O_4_ synthesized using urea. It appears that the particle size of ZnFe2O4 is slightly agglomerated. The particle size is between 10 and 20 nm, according to the results of calculations using XRD. Differences in particle size distribution can occur due to nonuniform heat during the combustion process.

## 4. Conclusion

The synthesis of ZnFe_2_O_4_ using the solution combustion method was conducted successfully. The several types of fuel used, namely urea, glycine, and EDTA, affected the physicochemical properties of the resulting ZnFe_2_O_4,_ which is characterized by a spinel structure. ZnFe_2_O_4_ synthesized using urea fuel has the smallest crystallite size and magnetic properties of 10.19 nm and 44.74 emu/g, but the largest surface area is 116.4 m^2^/g. Finally, the morphology of ZnFe_2_O_4_ synthesized using urea fuel appears to be more homogeneous than glycine and EDTA. The particle size of ZnFe_2_O_4_ was synthesized using urea in the range of 10–20 nm. These characteristics of ZnFe_2_O_4_ have the potential to be applied as adsorbent, catalyst and biomedical.

## Figures and Tables

**Table 1:** Data of X-ray diffraction.

Fuel	2θ (Degree)	Intensity (au)	d-spacing (Å)	Crystallite size (nm)
Urea	35.28	80.09	2.70	10.19
Glycine	35.26	318.08	2.98	20.34
EDTA	35.36	507.40	1.61	27.21

**Table 2:** Elements of ZnFe_2_O_4_.

Fuel	Zn (%)	Fe (%)	O (%)
Urea	26.89	46.55	25.56
Glycine	28.97	44.70	25.33
EDTA	30.16	43.55	25.29

**Table 3:** Crystallite size, surface area, and magnetic properties of ZnFe_2_O_4_ synthesized using several methods.

Synthesis method	Size (nm)	Surface area (m^2^/g)	Ms (emu/g)	Reference
Solid state method (ZnO, Fe_2_O_3_) variation calcination 900–1200 °C	51.9, 52.5, 53.0, and 53.4	-	-	[47]
Solution combustion (ratio: Zn: Fe: glycine= 1: 2:1.5)	15	40.3	11.9	[39]
Coprecipitation, ZnSO_4_.7H_2_O, FeSO_4_7H_2_O, and FeCl_3_	20	-	-	[48]
Lawsonia inermis leaf extract (Zn(CH_3_COO)_2_.2H_2_O and Fe(NO_3_)_3_.9H_2_O	17.12	-	42.93	[38]
Solution combustion (Fe(NO_3_)9H_2_O, Zn(NO_3_)_2_6H_2_O, aspartic acid, pH 10)	43	30.6		[49]
Solution combustion (ratio Zn:Fe = 1:2, triethylamine hydrochloride = 0.8, 1.0, 1.2, 1.4)	21; 25.4; 21.9 and 18.6	-	-	[50]
Coprecipitation (ZnO, Fe_2_O_3_ with variation sintering time (1.5, 2.5, and 3.5 h)	84.72; 70.58 and 84.72		1.12, 1.15, and 52.52	[51]
Moringa oleifera exctract (Fe(NO_3_)9H_2_O, Zn(NO_3_)_2_6H_2_O), annealed at 500 and 700 °C for 2 h	12.393, 16.076	-		[52]
Sol-gel method (FeCl_3_·6H_2_O, ZnCl_2_) with solvent EG, time reaction 2, 4 and 6 h	11.6. 16.2 and 20.5 nm		49.3, 53.8, and 61.3	[46]
Solution combustion (urea, glycine and EDTA)	10.19; 26.15 and 27.16	116.44, 100.6, and 94.2	44.74, 50.93 and 54.63	In this study
